# Clinical and laboratory features of childhood-onset primary Sjögren's syndrome: A retrospective study from China

**DOI:** 10.3389/fped.2022.1044812

**Published:** 2023-01-04

**Authors:** Chenxi Liu, Yingying Jin, Hua Huang, Fei Ding, Xuemei Xu, Shengfang Bao, Zhen Yang, Yanliang Jin

**Affiliations:** Department of Rheumatology and Immunology, Shanghai Children's Medical Center, Shanghai Jiao Tong University School of Medicine, Shanghai, China

**Keywords:** childhood-onset, primary Sjögren’s syndrome, clinical manifestation, laboratory examination, immunological markers

## Abstract

**Introduction:**

The initial presentations of childhood-onset primary Sjögren’s syndrome (C-pSS) vary, making diagnosis challenging. We aimed to improve the diagnosis and evaluation of C-pSS by summarizing its clinical and laboratory features.

**Methods:**

A total of 49 patients with C-pSS between July 2015 and August 2022 in the Department of Rheumatology and Immunology of Shanghai Children's Medical Centre were enrolled in this study. Their clinical manifestations and laboratory examinations of these patients were compared based on the presence or absence of thrombocytopenia and parotitis and whether the immunological markers, including anti-nuclear antibodies (ANA), rheumatoid factor (RF), anti-Ro52/SSA antibodies (anti-SSA/Ro52), anti-Ro60/SSA antibodies (anti-SSA/Ro60), and anti-Ro/SSB antibodies (anti-SSB), were positive.

**Results:**

The mean age at C-pSS diagnosis was 10.34 ± 3.45 years, and the ratio of boys to girls was 1:6. In the thrombocytopenia group, parotitis (*P *= 0.044), organ involvement except for hematology (*P *= 0.002), positive anti-SSB (*P *= 0.004), and positive RF (*P *= 0.001) were less frequently observed. Complement C4 (*P *= 0.038) and white blood cells (*P *= 0.002) levels decreased and increased significantly, respectively. Anti-SSB (*P *= 0.010) and RF (*P *= 0.004) positivity were independent potential protective factors against thrombocytopenia in patients with C-pSS. In the parotitis group, higher ANA titers (*P *= 0.027), higher focus scores on labial gland biopsy (*P *= 0.024), and positive RF (*P *= 0.001), anti-SSA/Ro60 (*P *= 0.003), and anti-SSB (*P *= 0.001) were observed more frequently. Furthermore, positive anti-SSB (*P *= 0.012) and positive RF (*P *= 0.028) were independent risk factors for parotitis in patients with C-pSS. The hemoglobin level was significantly lower in patients with positive anti-SSA/Ro52 and positive anti-SSA/Ro60 results (*P *= 0.022 and *P *= 0.029, respectively), while immunoglobulin G level was significantly higher in patients in the same group (*P *= 0.048 and *P *= 0.007, respectively).

**Conclusions:**

Positive anti-SSB and positive RF values may be independent potential protective factors of thrombocytopenia in patients with C-pSS. In contrast, positive anti-SSB and positive RF were independent risk factors of parotitis in patients with C-pSS. More studies are needed to reveal the diagnostic role and pathogenic mechanism of immunological markers in C-pSS.

## Introduction

Sjögren's syndrome (SS) is a chronic, systemic autoimmune disease. Nowadays, considering that the word “disease” can highlight the pathologic mechanisms of this kind of systemic rheumatic disease, and also make its treatment protocol easy to understand, some scholars prefer to call it Sjögren's disease rather than SS (1). Primary SS (pSS) refers to SS occurring without other connective tissue diseases; pSS is primarily characterized by decreased salivary and lacrimal gland function due to B-cell hyperactivity and lymphocytic infiltration but can also extend to multiple organs (2). In addition to typical clinical manifestations such as dry eyes and mouth, it can also present as caries, parotitis, arthritis, thrombocytopenia, leukopenia, interstitial lung disease (ILD), and renal tubular acidosis. The key immunological markers that may affect manifestation and prognosis include anti-Ro52/SSA antibodies (anti-SSA/Ro52), anti-Ro60/SSA antibodies (anti-SSA/Ro60), anti-Ro/SSB antibodies (anti-SSB), rheumatoid factor (RF), anti-nuclear antibodies (ANA), globulin (Glb), and immunoglobulin G (IgG) (3).

pSS mainly occurs in adults, with a peak age of approximately 50 years and a female-to-male ration of at least 9:1 (4). However, childhood-onset pSS (C-pSS) is rarely diagnosed at an early stage because its clinical manifestations are not typical and distinguishable enough, and no general diagnostic criteria can be referred to. These reasons even make some scholars misdiagnose it as a benign form of systemic lupus erythematosus (SLE) (5–8). However, with the increasing recognition and improvement of laboratory examinations for pSS, several large or multinational cohort studies based on pediatric populations have been reported. It was found that C-pSS involves approximately 1% of patients with pSS, with a male-to-female ratio of approximately 1:5–12 and a peak age of 10–14 years. Meanwhile, clinical phenotypes are dominated by parotid enlargement and systemic symptoms (7–12).

In addition, the initial presentations of C-pSS vary, making diagnosis challenging. In this study, we aim to improve the diagnosis and evaluation of C-pSS by summarizing its clinical and laboratory characteristics.

## Materials and methods

### Patients

Inpatients and outpatients aged younger than18 years with C-pSS who were initially diagnosed and being treated in the Department of Rheumatology and Immunology of Shanghai Children's Medical Centre between July 2015 and August 2022 were retrospectively analyzed. C-pSS was diagnosed according to the 2002 revised American-European Consensus Group (AECG) criteria (13), the American College of Rheumatology/European League Against Rheumatism (ACR/EULAR) criteria (14), the previously proposed diagnostic criteria for juvenile pSS (15), or the experience of pediatric rheumatologists. Children with other autoimmune diseases were excluded from this study. Clinical information for research purposes was approved by Shanghai Children's Medical Centre's Ethical Committee.

### Data collection

The features of the collected patients’ data were grouped into the following categories: demographics data (sex, age of onset, and diagnosis); clinical signs and symptoms at diagnosis (exocrine-glandular and extra-glandular); histological results and functional tests [labial gland (LSG) biopsy and Schirmer’s I test]; and laboratory/serological results [ANA, RF, anti-SSA/Ro52, anti-SSA/Ro60, anti-SSB, IgG, immunoglobulin A (IgA), immunoglobulin M (IgM), complement 3 (C3), complement 4 (C4), complement total activity (CH50), white blood cells (WBC), platelets (PLT), hemoglobin (Hb), erythrocyte sedimentation rate (ESR), liver function, kidney function, and cellular immune function]. For those patients treated more than once, we only collect and analyze their clinical and laboratory data from the first treatment, i.e., the data at the time of diagnosis.

Some of the definitions that needed to be specified are as follows: Parotitis groups were defined as recurrent episodes of symptomatic parotitis or parotid gland/submandibular gland abnormality indicated by ultrasound, including abnormal echogenicity of parenchymal tissue, inhomogeneous glands, the presence of hypoechogenic areas and hyperechogenic reflections, unclearness of salivary gland borders, and parotid duct dilation, while non-parotitis groups were defined as no recurrent parotitis history and abnormal ultrasounds; positive LSG biopsy was defined as focal lymphocytic sialadenitis with a focus score (FS) of ≥1 focus per 4 mm^2^, while a negative LSG biopsy was defined as the absence of focal lymphocytic sialadenitis; ILD was defined as a chronic and persistent cough, dyspnea, or pulmonary function test, which suggests decreased restrictive pulmonary ventilation or diffusing function, combined with nodules, ground-glass opacity, honeycombing, consolidation, septal thickening, and crazy paving in high-resolution computed tomography (HRCT); positive RF was defined as RF >15.9 IU/ml; leucopenia was defined as WBC <4 × 10^9^/L; thrombocytopenia was defined as PLT <100 × 10^9^/L; anemia was defined as Hb <110 g/L; hyperimmunoglobulin G was defined as IgG >16.2 g/L; hyperglobulin was defined as globulin > 32 g/L; and hypocomplementaemia was defined as C3 < 0.9 g/L or C4 < 0.1 g/L. An abnormal Schirmer’s I test result was defined as a score ≤ 5 mm/5 min; an abnormal tear film break-up time result was defined as a score <10 s; and an abnormal tear meniscus height result was defined as a score ≤ 0.35 mm. The ANA titer was tested by an indirect immunofluorescence assay (Euroimmun Medical Lab Diagnostics Stock Co., Lübeck, Germany), and a titer value > 1:100 was considered positive. The expressions of anti-SSA/Ro52, anti-SSA/Ro60, and anti-SSB were examined by Euroline ANA Profile IgG (Euroimmun Medical Lab Diagnostics Stock Co., Lübeck, Germany).

### Statistical analysis

SPSS (v.23.0) software was used for all data analyses. GraphPad Prism 7 was used for graph presentation. Categorical variables and continuous variables are described as numbers and percentages, and mean and standard deviation (SD), respectively. For between-group comparisons, the chi-square and Fisher exact tests were used for categorical variables, and two independent sample t-tests and Mann–Whitney U-tests were used for normally distributed and non-normally distributed continuous variables, respectively. Logistic regression analysis was used to explore the risk or protective factors for thrombocytopenia and parotitis in patients with pSS. Statistical significance was set at *P *<0.05.

## Results

### Patient characteristics

A total of 49 patients were included in this study. There were 35 (71.4%) patients who met the ACR/EULAR criteria, 12 (24.5%) who met the AECG criteria, and 31 (63.3%) who met the previously proposed diagnostic criteria for juvenile SS. About 6 (12.2%) patients met none of the criteria but were diagnosed by a pediatric rheumatologist (Supplementary Table S1). Of the 49 patients with pSS, 7 were boys and 42 were girls, with a male-to-female ratio of 1:6. The mean age at pSS onset was 9.50 ± 3.51 (range 3.33–15.83) years, and that at diagnosis was 10.34 ± 3.45 (range 3.33–16.42) years. The median time from pSS onset to diagnosis was 0.17 ± 1.53 (range 0–6.25) years.

Chief complaints of patients at first visit to department of rheumatology including the following: fever for 10 patients; thrombocytopenia for 21 patients; rash for 7 patients; gastrointestinal symptoms (belching, nausea, and hepatic dysfunction) for 4 patients; dry eyes and/or dry mouths for 2 patients; fatigue, anemia, alopecia areata, abnormal urine routine (microscopic hematuria for more than 6 years), and arthralgia for 1 patient, respectively. By further obtaining a detailed medical history from the patients and children, despite not presenting complaints, we found that 25 (51%) children had rashes of different types, with purpura being the most common; 14 (28.6%) experienced unexplained persistent or intermittent fever; 14 (28.6%) had multiple dental caries; 13 (26.5%) had arthritis or arthralgia; 13 (26.5%) had recurrent episodes of symptomatic parotitis; 9 (18.4%) had dry eyes and dry mouths; 8 (16.3%) had fatigue; and 3 (6.1%) had Raynaud's phenomenon. In addition, 32 (65.3%) patients had hematologic involvement, especially thrombocytopenia, of which the total number of patients was 22; 2 (4.1%) patients had renal involvement, of which 1 patient experienced renal tubular acidosis and 1 was diagnosed with mild glomerular lesions after renal biopsy. Forty-three patients underwent HRCT combined with pulmonary function test, of which 5 (11.6%) were considered to have lung involvement (4 had abnormal HRCT combined with abnormal lung function tests and 1 had abnormal HRCT combined with both abnormal pulmonary function tests and respiratory symptoms), among whom 1 patient experienced ILD and pulmonary hypertension concomitantly and 4 patients experienced ILD alone; 6 (12.2%) patients had digestive system involvement; 2 (4.1%) patients had musculoskeletal system involvement diagnosed by electromyography, suggesting myogenic damage; and only 1 (2%) patient who underwent repeated headache combined with cranial MRI, suggesting a few abnormal lesions in the bilateral frontal lobes, was diagnosed with central nervous system involvement.

For laboratory examination, among the 45 patients who underwent LSG biopsy, 39 (86.7%) were positive, and 31 could provide FS accurately, while 8 patients could not provide FS because of their limited memory and the long time since diagnosis. Schirmer's I test was performed on 43 patients, 20 (46.5%) of whom had abnormal results; a tear meniscus height examination was performed on 42 patients, 26 (61.9%) of whom had abnormal results; and a tear film break-up time examination was performed on 47 patients, 30 (63.8%) of whom had abnormal results. Among the 23 patients who were normal with Schirmer's I test, 8 were abnormal with tear film break-up time and 4 were abnormal with tear meniscus height. All patients were tested for ANA, anti-SSA/Ro52, anti-SSA/Ro60, and anti-SSB; 93.9% of which had positive ANA, 85.7% had positive anti-SSA/Ro52, 73.5% had positive anti-SSA/60, and 32.7% had positive anti-SSB. Meanwhile, 45 patients were tested for RF, and 16 (35.6%) had positive RF. In addition, a low C3 level was observed in 5 (5/47, 10.6%) patients, while a low C4 level was observed in 2 (2/47, 4.3%) patients; hyperimmunoglobulin G occurred in 21 (21/47, 44.7%) patients, while hyperglobulin occurred in 30 (61.2%) patients. When we divided these patients according to the diagnosis age (0–10 years groups and 10–18 years groups), there were no significant differences in either clinical or laboratory features (Table 1).

**Table 1:** Summary of clinical, laboratory, and histopathology features of childhood-onset pSS cohort (*n *= 49).

Features		Age at pSS diagnosis		* *
	Total	0 < age <10 (*n *= 21)	10 ≤ age < 18 (*n *= 28)	*P*-value
Demographics				
Sex (female/male)	42/7	18/3	24/4	1.000
Glandular manifestations				
Dry eyes (%)	18.4 (9/49)	23.8 (5/21)	14.3 (4/28)	0.632
Dry mouth (%)	18.4 (9/49)	19.0 (4/21)	17.9 (5/28)	1.000
Dental caries (%)	28.6 (14/49)	33.3 (7/21)	25.0 (7/28)	0.523
Parotitis (%)	50.0 (18/36)	47.1 (8/17)	52.6 (10/19)	1.000
Schirmer's I test (%)	46.5 (20/43)	50.0 (8/16)	44.4 (12/27)	0.724
LSG biopsy positive (%)	86.7 (39/45)	77.8 (14/18)	92.6 (25/27)	0.325
LSG biopsy FS ≥ 5 (%)	17.8 (8/45)	11.1 (2/18)	22.2 (6/27)	
LSG biopsy FS 1–4 (%)	51.1 (23/45)	44.4 (8/18)	55.6 (15/27)	
LSG biopsy positive but FS unclear (%)	17.8 (8/45)	22.2 (4/18)	14.8 (4/27)	
LSG biopsy negative (%)	13.3 (6/45)	22.2 (4/18)	7.4 (2/27)	
Extra-glandular manifestations				
Arthralgia or arthritis (%)	26.5 (13/49)	23.8 (5/21)	28.6 (8/28)	0.963
Fever (%)	28.6 (14/49)	23.8 (5/21)	32.1 (9/28)	0.523
Raynaud's phenomenon (%)	6.1 (3/49)	0 (0/21)	10.7 (3/28)	0.250
Rash (%)	51.0 (25/49)	66.7 (14/21)	39.3 (11/28)	0.058
Facial erythema (%)	10.2 (5/49)	19.0 (4/21)	3.6 (1/28)	
Chilblain (%)	2.0 (1/49)	0 (0/21)	3.6 (1/28)	
Urticaria (%)	2.0 (1/49)	4.8 (1/21)	0 (0/28)	
Purpura (%)	32.7 (16/49)	38.1 (8/21)	28.6 (8/28)	
Macula (%)	4.1 (2/49)	4.8 (1/21)	3.6 (1/28)	
Fatigue (%)	16.3 (8/49)	9.5 (2/21)	21.4 (6/28)	0.468
Hematology involvement (%)	65.3 (32/49)	66.7 (14/21)	64.3 (18/28)	0.862
Thrombocytopenia (%)	44.9 (22/49)	52.4 (11/21)	39.3 (11/28)	
Anemia (%)	10.2 (5/49)	9.5 (2/21)	10.7 (3/28)	
Leucopenia (%)	18.4 (9/49)	9.5 (2/21)	25.0 (7/28)	
Renal involvement (%)	4.1 (2/49)	0 (0/21)	7.1 (2/28)	0.500
Renal tubular acidosis (%)	2.0 (1/49)	0 (0/21)	3.6 (1/28)	
Mild glomerular lesions (%)	2.0 (1/49)	0 (0/21)	3.6 (1/28)	
Lung involvement (%)	11.6 (5/43)	10.5 (2/19)	12.5 (3/24)	1.000
Interstitial lung disease (%)	11.6 (5/43)	10.5 (2/19)	75.0 (3/4)	
Pulmonary hypertension (%)	2.3 (1/43)	0 (0/19)	25.0 (1/4)	
Digestive system involvement (%)	12.2 (6/49)	9.5 (2/21)	14.3 (4/28)	0.950
Hepatic dysfunction (%)	8.2 (4/49)	9.5 (2/21)	7.1 (2/28)	
Belching (%)	2.0 (1/49)	0 (0/21)	3.6 (1/28)	
Vomiting, abdominal pain and weight loss (%)	2.0 (1/49)	0 (0/21)	3.6 (1/28)	
Musculoskeletal system involvement (%)	4.1 (2/49)	4.8 (1/21)	3.6 (1/28)	1.000
Central nervous system involvement (%)	2.0 (1/49)	0 (0/21)	3.6 (1/28)	1.000
Continuing headache (%)	2.0 (1/49)	0 (0/21)	3.6 (1/28)	
Laboratory features				
Positive ANA (%)	93.9 (46/49)	95.2 (20/21)	92.9 (26/28)	1.000
ANA titer				0.195
≤1:100 (%)	32.7 (16/49)	23.8 (5/21)	39.3 (11/28)	
1:320 (%)	4.1 (2/49)	9.5 (2/21)	0 (0/28)	
≥1:1000 (%),	63.3 (31/49)	66.7 (14/21)	60.7 (17/28)	
Positive SSA/Ro52 (%)	85.7 (42/49)	90.5 (19/21)	82.1 (23/28)	0.680
ANA negative but SSA/Ro52+ (%)	2.4 (1/42)	5.3 (1/19)	0 (0/23)	
ANA titer1:100 and SSA/Ro52+ (%)	23.8 (10/42)	15.8 (3/19)	30.4 (7/23)	
ANA titer1:320 and SSA/Ro52+ (%)	4.8 (2/42)	10.5 (2/19)	0 (0/23)	
ANA titer ≥ 1:1000 and SSA/Ro52+ (%)	69.0 (29/42)	68.4 (13/19)	69.6 (16/23)	
Positive SSA/Ro60 (%)	73.5 (36/49)	76.2 (16/21)	71.4 (20/28)	0.709
ANA negative but SSA/Ro60+ (%)	0 (0/36)	0 (0/16)	0 (0/20)	
ANA titer1:100 and SSA/Ro60+ (%)	25.0 (9/36)	18.8 (3/16)	30.0 (6/20)	
ANA titer1:320 and SSA/Ro60+ (%)	2.8 (1/36)	6.3 (1/16)	0 (0/20)	
ANA titer ≥ 1:1000 and SSA/Ro60+ (%)	72.2 (26/36)	75.0 (12/16)	70.0 (14/20)	
Positive SSB (%)	32.7 (16/49)	33.3 (7/21)	32.1 (9/28)	0.930
ANA negative but SSB+ (%)	6.3 (1/16)	0 (0/7)	11.1 (1/9)	
ANA titer1:100 and SSB+ (%)	6.3 (1/16)	0 (0/7)	11.1 (1/9)	
ANA titer1:320 and SSB+ (%)	0 (0/16)	0 (0/7)	0 (0/9)	
ANA titer ≥ 1:1000 and SSB+ (%)	87.5 (14/16)	100.0 (7/7)	77.8 (7/9)	
Positive RF (%)	35.6 (16/45)	44.4 (8/18)	29.6 (8/27)	0.309

LSG, labial gland; FS, focus score; ANA, anti-nuclear antibodies; SSA/Ro52, anti-Ro52/SSA antibodies; SSA/Ro60, anti-Ro60/SSA antibodies; SSB, anti-Ro/SSB antibodies; RF, rheumatoid factor.

### Characteristics of pSS patients with or without thrombocytopenia

In this study, 22 (44.9%) patients experienced thrombocytopenia. Parotitis and organ involvement, except for hematology, occurred more frequently in the non-thrombocytopenia group than in the thrombocytopenia group (*P *= 0.044 and *P *= 0.002, respectively) (Table 2). The FS of LSG biopsy was lower in the thrombocytopenia group than in the non-thrombocytopenia group, although the difference was not significant (*P *= 0.118). Among pSS patients who underwent RF examination, only 1 (5.3%) patient in the thrombocytopenia group tested positive for RF, significantly less than the 15 (57.7%) patients in the control group (*P *= 0.001). Although there was no difference between the two groups, whether from the perspective of ANA positivity alone (*P *= 0.242) or even the titer of ANA (*P *= 0.536), patients with thrombocytopenia may still have a lower ANA titer. The presence of anti-SSA/Ro52 and anti-SSA/Ro60 was not significantly different between the two groups; however, when combined with ANA titers, we observed that patients in the thrombocytopenia group were more likely to have positive anti-SSA/Ro60 and ANA titers < 1:320 than those in the control group (*P *= 0.018). It is also worth noting that patients without thrombocytopenia were more likely to have positive anti-SSB than the thrombocytopenia group (*P *= 0.004) but had no relationship with the ANA titer (*P *= 1.000) (Table 2). Multiple regression analysis showed that the expressions of anti-SSB and positive RF were independent protective factors for thrombocytopenia in C-pSS patients (OR = 0.028, 95%CI 0.002–0.427, *P *= 0.010; OR = 0.020, 95%CI 0.001–0.291, *P *= 0.004, respectively) (Supplementary Table S2). Moreover, the level of C4 was significantly lower in the thrombocytopenia group (*P *= 0.038), while the levels of WBC and urea nitrogen (BUN) were significantly higher in the thrombocytopenia group (*P *= 0.002, and *P *= 0.001, respectively) (Table 2).

**Table 2:** Characteristics of pSS patients with or without thrombocytopenia and parotitis.

Parameters	pSS with thrombocytopenia (*n *= 22)	pSS without thrombocytopenia (*n *= 27)	*P*-value	pSS with parotitis (*n *= 18)	pSS without parotitis (*n *= 18)	*P*-value
Age (years) (mean ± SD)	10.03 ± 3.80	10.59 ± 3.20	0.576	10.63 ± 3.12	9.87 ± 3.83	0.516
Dry eyes (%)	18.2 (4/22)	18.5 (5/27)	1.000	16.7 (3/18)	16.7 (3/18)	1.000
Dry mouth (%)	18.2 (4/22)	18.5 (5/27)	1.000	27.8 (5/18)	22.2 (4/18)	1.000
Dental caries (%)	31.8 (7/22)	25.9 (7/27)	0.650	27.8 (5/18)	33.3 (6/18)	1.000
Parotitis (%)	29.4 (5/17)	68.4 (13/19)	**0**.**044**	**—**	**—**	**—**
Fever (%)	18.2 (4/22)	37.0 (10/27)	0.256	38.9 (7/18)	22.2 (4/18)	0.471
Arthritis or arthralgia (%)	27.3 (6/22)	25.9 (7/27)	0.915	22.2 (4/18)	27.8 (5/18)	1.000
Fatigue (%)	22.7 (5/22)	11.1 (3/27)	0.480	16.7 (3/18)	11.1 (2/18)	1.000
Raynaud's phenomenon (%)	9.1 (2/22)	3.7 (1/27)	0.855	11.1 (2/18)	5.6 (1/18)	1.000
Organ involvement except hematology (%)	22.7 (5/22)	66.7 (18/27)	**0**.**002**	**—**	**—**	**—**
Organ involvement (%)	**—**	**—**	** **	88.9 (16/18)	83.3 (15/18)	1.000
Schirmer's I test positive (%)	55.0 (11/20)	39.1 (9/23)	0.298	43.8 (7/16)	58.8 (10/17)	0.494
LSG biopsy performed			0.132			**0**.**024**
LSG biopsy FS 1–4 (%)	85.7 (12/14)	58.8 (10/17)		40.0 (4/10)	90.9 (10/11)	
LSG biopsy ≥5 (%)	14.3 (2/14)	41.2 (7/17)		60.0 (6/10)	9.0 (1/11)	
IgG (g/L)	17.99 ± 8.36	16.21 ± 5.71	0.495	19.30 ± 8.29	15.73 ± 7.08	0.176
IgA (g/L)	1.68 ± 0.73	2.08 ± 0.87	0.093	2.12 ± 0.71	1.91 ± 0.99	0.471
IgM (g/L)	1.08 ± 0.46	1.34 ± 0.56	0.054	1.44 ± 0.59	1.10 ± 0.40	0.075
C3 (g/L)	1.16 ± 0.18	1.16 ± 0.32	0.398	1.18 ± 0.31	1.22 ± 0.24	0.391
C4 (g/L)	0.17 ± 0.06	0.23 ± 0.10	**0**.**038**	0.22 ± 0.10	0.20 ± 0.09	0.487
CH50 (U/ml)	39.67 ± 12.85	37.80 ± 13.40	0.592	40.56 ± 10.91	41.47 ± 12.13	0.576
ALT (U/L)	32.73 ± 25.2	32.48 ± 27.09	0.802	33.72 ± 28.42	29.72 ± 18.36	0.825
AST (U/L)	31.95 ± 14.84	32.44 ± 16.70	0.944	34.11 ± 18.66	29.89 ± 10.80	0.800
Globulin (g/L)	34.90 ± 7.22	33.74 ± 5.74	0.615	36.02 ± 7.41	32.59 ± 6.03	0.138
WBC (×10^9^/L)	8.29 ± 3.20	5.83 ± 2.03	**0**.**002**	6.17 ± 2.16	8.25 ± 3.54	0.114
Hb (g/L)	123.77 ± 17.85	121.59 ± 19.66	0.644	117.67 ± 20.85	123.72 ± 21.30	0.569
Cr (μmol/L)	39.55 ± 9.70	42.67 ± 9.92	0.274	39.22 ± 8.16	42.45 ± 10.65	0.329
BUN (mmol/L)	4.97 ± 0.83	4.18 ± 0.81	**0**.**001**	4.39 ± 0.99	4.61 ± 0.86	0.486
CD3-/CD19+ (cells/μl)	639.48 ± 515.48	556.11 ± 488.60	0.222	542.93 ± 544.36	649.28 ± 469.80	0.228
ESR (mm/h)	16.38 ± 15.38	22.52 ± 26.13	0.408	28.53 ± 32.49	15.45 ± 12.97	0.223
Hyperimmunoglobulinaemia (%)	59.1 (13/22)	32 (8/25)	0.062	77.8 (14/18)	38.9 (7/18)	**0**.**041**
Hyperglobulin (%)	63.6 (14/22)	59.3 (16/27)	0.754	58.8 (10/18)	44.4 (8/18)	0.740
Positive RF (%)	5.3 (1/19)	57.7 (15/26)	**0**.**001**	62.5 (10/16)	5.6 (1/18)	**0**.**001**
Positive ANA (%)	100.0 (22/22)	88.9 (24/27)	0.242	100.0 (18/18)	88.9 (16/18)	0.486
ANA titer			0.536			**0**.**027**
≤1:100 (%)	40.9 (9/22)	25.9 (7/27)		11.1 (2/18)	50.0 (9/18)	
1:320 (%)	4.5 (1/22)	3.7 (1/27)		5.6 (1/18)	5.6 (1/18)	
≥1:1000 (%)	54.5 (12/22)	70.4 (19/27)		83.3 (15/18)	44.4 (8/18)	
Positive SSA/Ro52 (%)	86.4 (19/22)	85.2 (23/27)	1.000	100.0 (18/18)	77.8 (14/18)	0.104
ANA titer < 1:320 and SSA/Ro52+ (%)	36.8 (7/19)	17.4 (4/23)	0.283	11.1 (2/18)	50.0 (7/14)	**0**.**022**
ANA titer ≥ 1:320 and SSA/Ro52+ (%)	63.2 (12/19)	82.6 (19/23)		88.9 (16/18)	50.0 (7/14)	** **
Positive SSA/Ro60 (%)	63.6 (14/22)	81.5 (22/27)	0.159	100.0 (18/18)	55.6 (10/18)	**0**.**003**
ANA titer < 1:320 and SSA/Ro60+ (%)	50.0 (7/14)	9.1 (2/22)	**0**.**018**	11.1 (2/18)	50.0 (5/10)	0.063
ANA titer ≥ 1:320 and SSA/Ro60+ (%)	50.0 (7/14)	90.9 (20/22)	** **	88.9 (16/18)	50.0 (5/10)	
Positive SSB (%)	9.1 (2/22)	51.9 (14/27)	**0**.**004**	61.1 (11/18)	5.6 (1/18)	**0**.**001**
ANA titer < 1:320 and SSB+ (%)	0 (0/2)	7.1 (1/14)	1.000	0 (0/11)	100.0 (1/1)	**—**
ANA titer ≥ 1:320 and SSB+ (%)	100.0 (2/2)	92.9 (13/14)		100 (11/11)	0 (0/1)	

LSG, labial gland; FS, focus score; ANA, anti-nuclear antibodies; SSA/Ro52, anti-Ro52/SSA antibodies; SSA/Ro60, anti-Ro60/SSA antibodies; SSB, anti-Ro/SSB antibodies; RF, rheumatoid factor; C3, complement 3; C4, complement 4; CH50, complement total activity; IgG, immunoglobulin G; IgA, immunoglobulin A; IgM, immunoglobulin M; ESR, erythrocyte sedimentation rate; WBC, white blood cells; Hb, hemoglobin; PLT, platelets; BUN, urea nitrogen; ALT, alanine aminotransferase; AST, aspartate aminotransferase; Cr, creatinine; ESR, erythrocyte sedimentation rate. The bold values are the values with statistical significance.

### Characteristics of pSS patients with or without parotitis

For parotitis, we only take 36 ultrasound-checked patients into account and partition them into two groups. In these 36 patients, 9 were reported to have parotitis by both abnormal ultrasounds and recurrent parotitis history, 5 only by abnormal ultrasounds, and 4 only by recurrent parotitis history. Thus, 18 patients were divided into the parotitis group, and the other 18 patients were divided into the non-parotitis group. The parotitis group had significantly higher FS than the control group (*P *= 0.024). There was no significant difference in ANA expression because 18 patients with parotitis and 16 patients without parotitis tested positive for ANA (*P *= 0.486); however, when we compared the difference according to the ANA titer, we discovered that patients with parotitis had a higher ANA titer (*P *= 0.027). Eighteen patients with parotitis were anti-SSA/Ro60 positive, and 11 patients with parotitis were anti-SSB positive, compared with 14 and 1 patients without parotitis who were anti-SSA/Ro60 positive or anti-SSB positive, respectively, and the differences were statistically significant (*P *= 0.003 and *P *= 0.001, respectively). Among pSS patients who underwent RF examination, 10 (62.5%) patients in the parotitis group tested positive for RF, which was significantly higher than 1 (5.6%) in the control group (*P *= 0.001) (Table 2). Furthermore, multiple logistic regression analysis revealed that SSB and RF positivity were independent risk factors for parotitis in patients with pSS (OR = 30.518, 95%CI 2.102–443.006, *P *= 0.012; and OR = 20.451, 95%CI 1.393–300.207, *P *= 0.028, respectively) (Supplementary Table S3).

### Comparison of the effect of ANA, SSA/Ro52, SSA/60, SSB, and RF on organ involvement and clinical or other laboratory features

Patients with negative RF results were more likely to experience hematological involvement than those with RF positivity (*P *= 0.003). However, there were no significant differences in other organ involvement between the immunological markers of pSS (Supplementary Table S4). Although the frequency of lung involvement in patients with positive anti-SSA/Ro52 results was higher than that in those who tested negative for anti-SSA/Ro52, the difference was not statistically significant (13.5% vs. 0%, *P *= 1.000).

**Figure 1:**
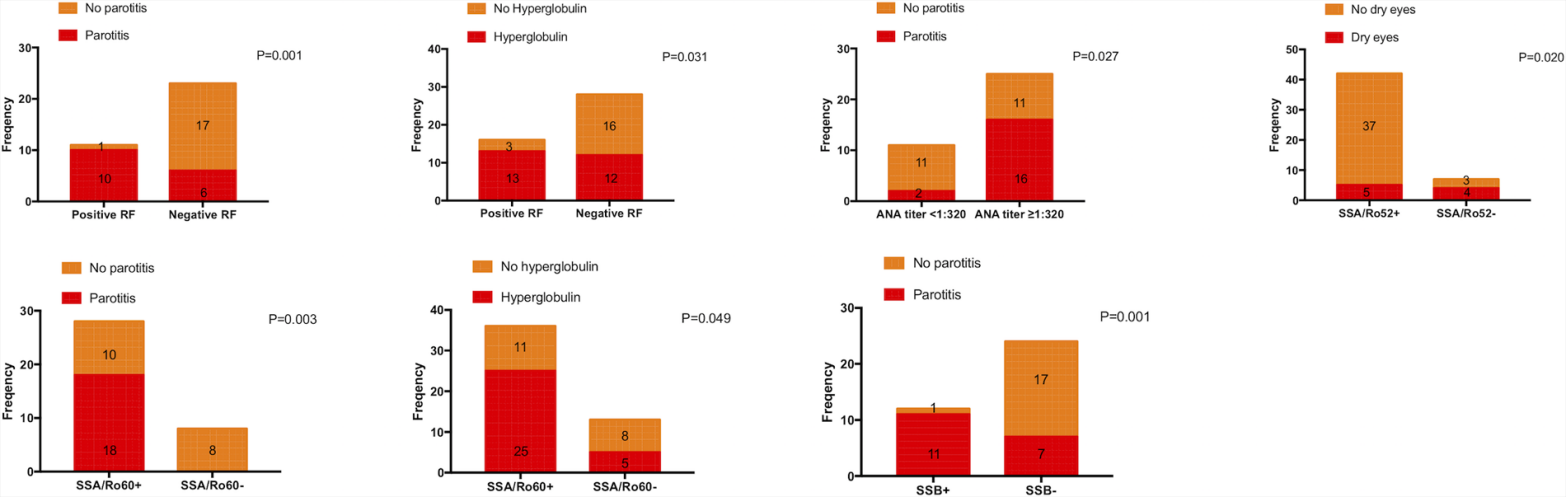
Clinical features associated with patients with positive SSA/Ro52, positive SSA/Ro60, and positive SSB. ANA, anti-nuclear antibodies; SSA/Ro52, anti-Ro52/SSA antibodies; SSA/Ro60, anti-Ro60/SSA antibodies; SSB, anti-Ro/SSB antibodies; RF, rheumatoid factor.

**Figure 2:**
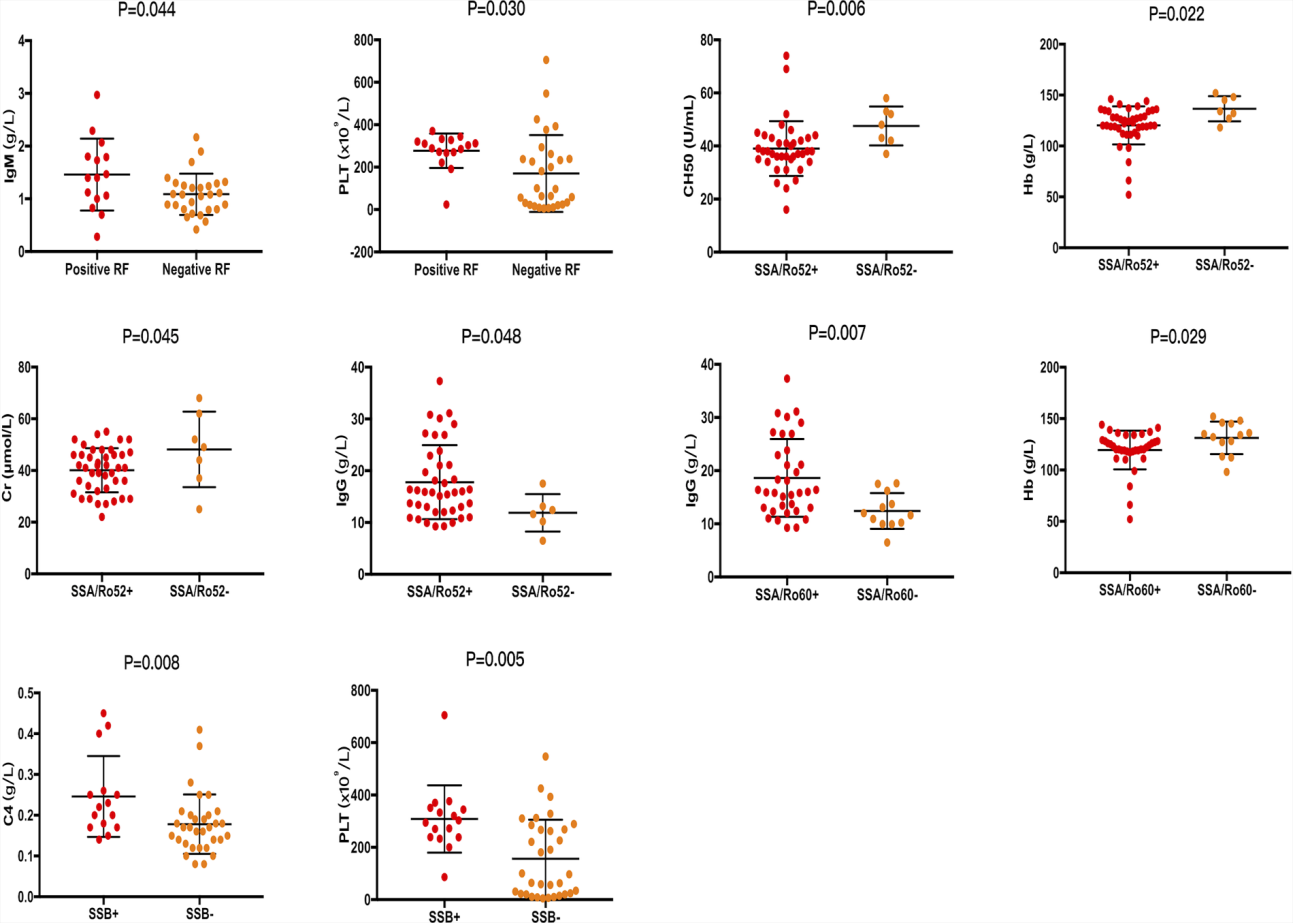
Laboratory features associated with patients with positive SSA/Ro52, positive SSA/Ro60, and positive SSB. ANA, anti-nuclear antibodies; SSA/Ro52, anti-Ro52/SSA antibodies; SSA/Ro60, anti-Ro60/SSA antibodies; SSB, anti-Ro/SSB antibodies; RF, rheumatoid factor; C4, complement 4; CH50, complement total activity; IgG, immunoglobulin G; IgM, immunoglobulin M; Hb, hemoglobin; PLT, platelets; Cr, creatinine.

Consistent with the conclusions we have mentioned above, patients with positive RF, ANA titer ≥1:320, positive anti-SA/Ro60, and positive anti-SSB were more likely to experience parotitis (*P *= 0.001, *P *= 0.027, *P *= 0.003, and *P *= 0.001, respectively). Moreover, dry eyes were more likely to occur in patients with a negative anti-SSA/Ro52 (*P *= 0.020), and hyperglobulin was more likely to occur in patients with positive RF and anti-SSA/Ro60 (*P *= 0.031 and *P *= 0.049, respectively) (Figure 1). In addition, the PLT value was higher in patients with positive RF and anti-SSB (*P *= 0.030, and *P *= 0.005, respectively), the Hb value was lower in patients with positive anti-SSA/Ro52 and anti-SSA/Ro60 (*P *= 0.022, and *P *= 0.029, respectively), and the level of IgG was higher in patients with positive anti-SSA/Ro52 and anti-SSA/Ro60 (*P *= 0.048, and *P *= 0.007, respectively) (Figure 2). Meanwhile, positive RF was more common in patients with positive SSA/Ro52 and SSA/Ro60 results (*P *= 0.040 and *P *= 0.001, respectively).

## Discussion

To our knowledge, this is the first single-cohort retrospective study on pSS in Chinese children. The female-to-male ratio of C-pSS in our study was 6:1, and the mean age at pSS diagnosis was 10.34 ± 3.45 (range 3.33–16.42) years, consistent with previous child cohort studies (7–12). However, the existing diagnostic criteria for adult pSS patients do not apply to children, which has been confirmed by other scholars (7, 10, 16, 17). In our hospital, the examination of unstimulated whole salivary flow, salivary scintigraphy, and parotid sialography had not been developed and promoted yet. Under these conditions, only 71.4% of patients met the ACR/EULAR criteria, 24.5% of patients met the AECG criteria, and 63.3% of patients met the previously proposed diagnostic criteria for juvenile SS. Combined with cases in our hospital and reported literature, we present some reasons why existing diagnostic criteria may be inadequate for children. First, several recent large or multicenter cohort studies found that among C-pSS patients, parotitis was the most common symptom at presentation, although dry mouth and dry eyes were commonly reported, and systemic symptoms, including unexplained fever, arthralgia, dental caries, and organ involvement were more likely to happen (8–12). Consistently, the most common manifestation of pSS in our study was rash (51%), especially purpura, followed by parotitis (36.7%), whereas hematology was the most common organ (65.3%). However, all of these symptoms were excluded from the diagnostic criteria for adults. Second, other ophthalmic examinations, such as tear meniscus height and tear film break-up time, which are clinically convenient to perform but are not included in the diagnostic criteria, are also useful for the diagnosis of pSS (18). In our hospital, these three ophthalmic examinations were commonly performed simultaneously. Among the 23 patients who were normal with Schirmer's I test, 8 were abnormal with tear film break-up time, and 4 were abnormal with tear meniscus height. Third, six patients in our study met the previously proposed diagnostic criteria for juvenile SS when laboratory indications were added, emphasizing the importance of serological evaluation in pSS diagnosis in children. Fourth, unstimulated whole salivary flow, salivary scintigraphy, and parotid sialography can be abnormal in children and may provide sufficient evidence for a C-pSS diagnosis (17, 19); however, patients who do not exhibit parotitis at the hospital visit may refuse to be tested. Notably, as a non-invasive examination to assess parotid gland involvement, salivary gland ultrasonography was recently demonstrated to be effective in C-pSS diagnosis (20–22). Therefore, to reduce missed pSS diagnoses in children, we suggest that all suspected patients undergo a parotid gland ultrasound examination.

We observed that thrombocytopenia occurred in 22 (44.9%) patients, higher than 12.03%–23.9% reported by a few studies based on adult pSS patients (23–25), but similar to the 48.9% reported by Wu et al. (26). However, published pediatric cohort studies focusing on thrombocytopenia secondary to pSS are lacking at present. In fact, the detailed mechanisms of platelet destruction have not yet been fully elucidated, although thrombocytopenia is often secondary to autoimmune diseases. Previous studies have mainly focused on plasma *P*-selectin autoantibodies (27), whereas recent studies have focused on B-cell activating factors and Toll-like receptors (28), suggesting that humoral immune response plays an important role in platelet destruction in pSS. In our study, although the difference was not statistically significant, we observed that the level of CD3−/CD19 + cells in the peripheral blood of patients with thrombocytopenia tended to be higher than that in patients without thrombocytopenia, indicating that B cells may be expanded in patients with thrombocytopenia. However, the exact conclusion needs to be confirmed in future cell and animal experiments. Moreover, we observed that the incidence of parotitis was significantly lower in patients with thrombocytopenia than in non-thrombocytopenia patients. Similarly, patients with thrombocytopenia appeared to have a lower FS on LSG biopsies, even though no significant difference was observed in both groups. This suggests that pSS patients with thrombocytopenia may be less likely to experience exocrine gland involvement. Reportedly, ILD is negatively related to thrombocytopenia in pSS (23, 24, 26); however, due to the limited number of patients with ILD in our study, we analyzed the comparison of organ involvement, except that of hematology, and found that patients with thrombocytopenia were less likely to experience other organ involvement. Moreover, autoantibodies inducing complement-mediated destruction of platelets and inhibiting megakaryocyte function are reportedly involved in thrombocytopenia development (29, 30). Several studies have also shown that complement levels were significantly reduced in patients with thrombocytopenia, and a low C4 level could predict more severe thrombocytopenia (23, 25, 26, 31). Our results also suggested that low C4 levels may seriously influence the development of thrombocytopenia in patients with C-pSS. Finally, we observed that SSB and RF were negatively associated with thrombocytopenia in pSS, which is consistent with the conclusions of previous studies (23, 25, 26). However, more research is needed to verify these findings and explore the underlying mechanisms.

Because parotitis may be one of the most common symptoms of C-pSS and studies have also demonstrated that parotitis is negatively associated with age, even in children with pSS (7, 10, 12), we analyzed the characteristics of patients with or without parotitis. However, we observed that there were errors in the medical history provided because the family members had insufficient understanding of the clinical symptoms of parotitis, or the child was relatively young and had no chief complaint of intense parotid gland swelling and pain. Therefore, we combined the results of the ultrasound examination before grouping. We observed that a higher FS of LSG biopsy, ANA titer ≥ 1:320, positive RF, anti-SSA/Ro60, and anti-SSB values were more likely to occur in patients with parotitis, and that positive RF and anti-SSB were independent risk factors for parotitis in pSS. These results indicated a strong association between autoantibody positivity and histopathological changes in pSS and showed that lymphocytic infiltration and autoantibody production might be early features in the immunopathogenesis with subsequent gland dysfunction and end-organ damage. Similar results showing that pSS patients with RF and anti-SSA positivity were more likely to experience parotitis were also reported by other studies (7, 32–34); however, one study reported that anti-SSA was common in patients without parotitis (10), which was inconsistent with our study. However, to date, no more studies have focused on the mechanisms of immunological biomarkers and parotitis in patients with C-pSS.

In addition to focusing on clinical manifestations, we also analyzed traditional immunological markers. First, the positive proportions of ANA, anti-SSA, anti-SSB, and RF were 88%, 74%, 45%, and 60%, respectively, in C-pSS patients, as reported by an international cohort study (10). In our study, the positive proportions of ANA, anti-SSA/Ro52, anti-SSA/Ro60, anti-SSB, and RF were 93.9%, 85.7%, 73.5%, 32.7%, and 35.6%, respectively. Meanwhile, an ANA titer ≥1:320, which reached the diagnostic standard of pSS for adults, was observed in 67.4% of the patients. Second, organ involvement has been associated with immunological markers. Gao et al. reported RF was the independent predictors of ILD in pSS patients (35), while Gao et al., Davidson et al., and Yazisiz et al. have previously shown that pSS patients with positive anti-SSA were more likely to experience ILD (35–37). However, except for the negative correlation between positive RF and hematologic involvement, no other significant differences were observed in our study. This may be related to the limited number of patients with C-pSS and the relatively low probability of organ involvement in our study. Third, a close association between immunological markers and other laboratory indications identified in our study has also been reported in previous studies (32, 34, 38). We confirmed that IgG levels increased significantly in patients with positive anti-SSA/Ro52 or anti-SSA/Ro60. One possible hypothesis is that the interaction of autoantibodies with the autoantigens can cause chronic inflammation, followed by the production of various cytokines and chemokines, which can lead to B-cell activation and proliferation. Then, plasma cells differentiated by activated B cells can produce various autoantibodies, including RF and polyclonal immunoglobulins (39). However, it is worth noting that the C4 level was lower in patients with negative SSB values and thrombocytopenia, while patients with negative anti-SSB were more likely to develop thrombocytopenia, suggesting a possible but unclear relationship between anti-SSB, C4, and thrombocytopenia.

However, some limitations of this study cannot be ignored. First, as a single-cohort retrospective study, the sample size was relatively small. Second, we mainly focused on the clinical and laboratory features; thus, we did not summarize the treatment therapy and prognosis of C-pSS. Third, only children's cohorts were included in this study. However, to identify more unique characteristics of C-pSS, a control cohort comprising adults will be required in the future.

In conclusion, we observed that the chief complaints and clinical symptoms varied in patients with C-pSS, thus establishing that pediatric-specific criteria are essential for diagnosing pSS in children. Moreover, positive anti-SSB and RF may be independent potential protective factors for thrombocytopenia in pSS. To investigate whether patients with new onset thrombocytopenia are secondary to pSS or not, we should also refer to the examination results of ANA, C3, C4, IgG, and LSG biopsy. Immunological markers may play a vital role in parotitis development in pSS, as positive RF and anti-SSB were independent risk factors for parotitis in pSS. In the future, more studies are needed to investigate the diagnostic role and pathogenic mechanism of immunological markers in C-pSS.

## Data Availability

The original contributions presented in the study are included in the article/Supplementary Material, further inquiries can be directed to the corresponding author.

## References

[1] Baer AN Hammitt KM. Sjögren's disease, not syndrome. Arthritis Rheumatol. (2021) 73(7):1347–8. 10.1002/art.41676 33559389

[2] Mavragani CP. Mechanisms and new strategies for primary Sjögren’s syndrome. Annu Rev Med. (2017) 68:331–43. 10.1146/annurev-med-043015-123313 28099084

[3] Jonsson R Brokstad KA Jonsson MV Delaleu N Skarstein K. Current concepts on Sjögren’s syndrome-classification criteria and biomarkers. Eur J Oral Sci. (2018) 126(Suppl 1):37–48. 10.1111/eos.12536 30178554 PMC6586012

[4] Qin B Wang J Yang Z Yang M Ma N Huang F Epidemiology of primary Sjögren’s syndrome: a systematic review and meta-analysis. Ann Rheum Dis. (2015) 74(11):1983–9. 10.1136/annrheumdis-2014-205375 24938285

[5] Taşdemir M Hasan C Ağbaş A Kasapçopur Ö Canpolat N Sever L Sjögren’s syndrome associated with systemic lupus erythematosus. Turk Arch Pediatr. (2016) 51(3):166–8. 10.5152/TurkPediatriArs.2016.2001 PMC5047367 27738403

[6] Heaton JM. Sjögren’s syndrome and systemic lupus erythematosus. Br Med J. (1959) 1(5120):466–9. 10.1136/bmj.1.5120.466 13629021 PMC1992781

[7] Yokogawa N Lieberman SM Sherry DD Vivino FB. Features of childhood Sjögren’s syndrome in comparison to adult Sjögren’s syndrome: considerations in establishing child-specific diagnostic criteria. Clin Exp Rheumatol. (2016) 34(2):343–51. 26812559

[8] Theander E Jacobsson LT. Relationship of Sjögren’s syndrome to other connective tissue and autoimmune disorders. Rheum Dis Clin North Am. (2008) 34(4):935–47. 10.1016/j.rdc.2008.08.009 18984413

[9] Ramos-Casals M Acar-Denizli N Vissink A Brito-Zerón P Li X Carubbi F Childhood-onset of primary Sjögren’s syndrome: phenotypic characterization at diagnosis of 158 children. Rheumatology. (2021) 60(10):4558–67. 10.1093/rheumatology/keab032 33493333

[10] Basiaga ML Stern SM Mehta JJ Edens C Randell RL Pomorska A Childhood Sjögren syndrome: features of an international cohort and application of the 2016 ACR/EULAR classification criteria. Rheumatology. (2021) 60(7):3144–55. 10.1093/rheumatology/keaa757 33280020 PMC8487648

[11] Kaleda MI Nikishina IP Latypova AN. Sjögren’s syndrome with juvenile onset. Ter Arkh. (2019) 91(5):54–60. 10.26442/00403660.2019.05.000189 32598677

[12] Marino A Romano M Giani T Gaggiano C Costi S Singh R Childhood Sjogren’s syndrome: an Italian case series and a literature review-based cohort. Semin Arthritis Rheum. (2021) 51(4):903–10. 10.1016/j.semarthrit.2020.11.004 33261821

[13] Vitali C Bombardieri S Jonsson R Moutsopoulos HM Alexander EL Carsons SE Classification criteria for Sjögren’s syndrome: a revised version of the European criteria proposed by the American-European consensus group. Ann Rheum Dis. (2002) 61(6):554–8. 10.1136/ard.61.6.554 12006334 PMC1754137

[14] Shiboski CH Shiboski SC Seror R Criswell LA Labetoulle M Lietman TM 2016 American College of Rheumatology/European League against rheumatism classification criteria for primary Sjögren’s syndrome: a consensus and data-driven methodology involving three international patient cohorts. Ann Rheum Dis. (2017) 76(1):9–16. 10.1136/annrheumdis-2016-210571 27789466

[15] Bartůnková J Sedivá A Vencovský J Tesar V. Primary Sjögren’s syndrome in children and adolescents: proposal for diagnostic criteria. Clin Exp Rheumatol. (1999) 17(3):381–6. 10410277

[16] Houghton K Malleson P Cabral D Petty R Tucker L. Primary Sjögren’s syndrome in children and adolescents: are proposed diagnostic criteria applicable? J Rheumatol. (2005) 32(11):2225–32. 16265707

[17] Pomorska A Świętoń D Lieberman SM Bryl E Kosiak W Pęksa R Recurrent or persistent salivary gland enlargement in children: when is it Sjögren’s? Semin Arthritis Rheum. (2022) 52:151945. 10.1016/j.semarthrit.2021.11.011 35000785

[18] Sumida T Azuma N Moriyama M Takahashi H Asashima H Honda F Clinical practice guideline for Sjögren’s syndrome 2017. Mod Rheumatol. (2018) 28(3):383–408. 10.1080/14397595.2018.1438093 29409370

[19] Tomiita M Ueda T Nagata H Tanabe E Shimojo N Saito K Usefulness of magnetic resonance sialography in patients with juvenile Sjögren’s syndrome. Clin Exp Rheumatol. (2005) 23(4):540–4. 16095127

[20] Hammenfors DS Valim V Bica BERG Pasoto SG Lilleby V Nieto-González JC Juvenile Sjögren’s syndrome: clinical characteristics with focus on salivary gland ultrasonography. Arthritis Care Res. (2020) 72(1):78–87. 10.1002/acr.23839 PMC6972604 30697959

[21] Krumrey-Langkammerer M Haas JP. Salivary gland ultrasound in the diagnostic workup of juvenile Sjögren’s syndrome and mixed connective tissue disease. Pediatr Rheumatol Online J. (2020) 18(1):44. 10.1186/s12969-020-00437-6 32517804 PMC7285617

[22] Silva JL Faria DS Neves JS Cerqueira M Peixoto D Teixeira F. Salivary gland ultrasound findings are associated with clinical and serologic features in primary Sjögren’s syndrome patients. Salivary gland ultrasound findings are associated with clinical and serologic features in primary Sjögren’s syndrome patients. Acta Rheumatol Port. (2020) 45(1):76–7. 32608383

[23] Luo J Song WJ Chen JQ Yang GY Yang JY Yu XB Factors associated with secondary immune thrombocytopenia in patients with primary Sjögren’s syndrome: a retrospective study of 639 cases. Clin Exp Rheumatol. (2022). 10.55563/clinexprheumatol/8hgmjm. [Epub ahead of print] 35383565

[24] Koh JH Lee J Chung SH Kwok SK Park SH. Relation of autoimmune cytopenia to glandular and systemic manifestations in primary Sjögren syndrome: analysis of 113 Korean patients. J Rheumatol. (2015) 42(10):1817–24. 10.3899/jrheum.150058 26276967

[25] Dai F Yang G Rao P Wu P Chen R Sun Y Clinical characteristics of secondary immune thrombocytopenia associated with primary Sjögren’s syndrome. Front Med. (2020) 7:138. 10.3389/fmed.2020.00138 PMC7181055 32363196

[26] Wu J Chang X Zhang J Liu C Liu M Chen W. Clinical and laboratory features of primary Sjögren’s syndrome complicated with mild to severe thrombocytopenia. Ann Transl Med. (2022) 10(6):300. 10.21037/atm-22-162 35433982 PMC9011250

[27] Hu YH Zhou PF Long GF Tian X Guo YF Pang AM Elevated plasma P-selectin autoantibodies in primary Sjögren syndrome patients with thrombocytopenia. Med Sci Monit. (2015) 21:3690–5. 10.12659/msm.895144 26613867 PMC4668912

[28] Zhang S Qu J Wang L Li M Xu D Zhao Y Activation of toll-like receptor 7 signaling pathway in primary Sjögren’s syndrome-associated thrombocytopenia. Front Immunol. (2021) 12:637659. 10.3389/fimmu.2021.637659 33767707 PMC7986855

[29] Audia S Mahévas M Samson M Godeau B Bonnotte B. Pathogenesis of immune thrombocytopenia. Autoimmun Rev. (2017) 16(6):620–32. 10.1016/j.autrev.2017.04.012 28428120

[30] Cooper N Ghanima W. Immune thrombocytopenia. N Engl J Med. (2019) 381(10):945–55. 10.1056/NEJMcp1810479 31483965

[31] Cheloff AZ Kuter DJ Al-Samkari H. Serum complement levels in immune thrombocytopenia: characterization and relation to clinical features. Res Pract Thromb Haemost. (2020) 4(5):807–12. 10.1002/rth2.12388 32685889 PMC7354388

[32] Retamozo S Akasbi M Brito-Zerón P Bosch X Bove A Perez-de-Lis M Anti-Ro52 antibody testing influences the classification and clinical characterisation of primary Sjögren’s syndrome. Clin Exp Rheumatol. (2012) 30(5):686–92. 22704838

[33] Routsias JG Tzioufas AG. Autoimmune response and target autoantigens in Sjogren’s syndrome. Eur J Clin Invest. (2010) 40(11):1026–36. 10.1111/j.1365-2362.2010.02342.x 20629708

[34] Zhao Y Li Y Wang L Li XF Huang CB Wang GC Primary Sjögren syndrome in Han Chinese: clinical and immunological characteristics of 483 patients. Medicine. (2015) 94(16):e667. 10.1097/MD.0000000000000667 25906094 PMC4602699

[35] Gao H Zhang XW He J Zhang J An Y Sun Y Prevalence, risk factors, and prognosis of interstitial lung disease in a large cohort of Chinese primary Sjögren syndrome patients: a case-control study. Medicine. (2018) 97(24):e11003. 10.1097/MD.0000000000011003 29901591 PMC6023797

[36] Yazisiz V Arslan G Ozbudak IH Turker S Erbasan F Avci AB Lung involvement in patients with primary Sjögren’s syndrome: what are the predictors? Rheumatol Int. (2010) 30(10):1317–24. 10.1007/s00296-009-1152-8 19844720

[37] Davidson BK Kelly CA Griffiths ID. Ten year follow up of pulmonary function in patients with primary Sjögren’s syndrome. Ann Rheum Dis. (2000) 59(9):709–12. 10.1136/ard.59.9.709 10976085 PMC1753261

[38] Nakamura H Morimoto S Shimizu T Takatani A Nishihata SY Kawakami A. Clinical manifestations in anti-Ro52/SS-A antibody-seropositive patients with Sjögren’s syndrome. Immunol Med. (2021) 44(4):252–62. 10.1080/25785826.2021.1919342 33989125

[39] Psianou K Panagoulias I Papanastasiou AD de Lastic AL Rodi M Spantidea PI Clinical and immunological parameters of Sjögren’s syndrome. Autoimmun Rev. (2018) 17(10):1053–64. 10.1016/j.autrev.2018.05.005 30103041

