# Supplementary material for: Clinical and laboratory features of childhood-onset primary Sjögren's syndrome: A retrospective study from China
Source: Front Pediatr. 2023 Jan 4;10:1044812. doi: 10.3389/fped.2022.1044812 (PMC9846242; doi:10.3389/fped.2022.1044812)
Supplement: Supplementary file 1 [file Table1.docx]

| **Supplemental Table S1** Cases diagnosed by the experience of pediatric rheumatologist | | | | |
| --- | --- | --- | --- | --- |
| Case | Age at diagnosis | Sex | Clinical manifestations | Laboratory features |
| 1 | 16.42 | Male | Dry eyes, dry mouth, caries, oral ulcer | TMH: OD 0.12mm/ OS 0.14mm, conjunctivitis |
| 2 | 8.75 | Female | Facial erythema, fever, arthralgia | ANA 1:1000, SSA/Ro52 (+), SSA/Ro60 (+), SSB (+), RF (+), BUT: OD 6.17s/OS 6.50s |
| 3 | 9 | Female | Caries, dry eyes | LGS: FS=1, TMH: OD 0.11mm/ OS 0.14mm, ANA 1:100, thrombocytopenia, hyperglobulin |
| 4 | 5.25 | Female | Caries, dry eyes | LGS: FS=1, ANA 1:1000, thrombocytopenia |
| 5  6 | 7.75  10 | Female  Female | Dry mouth  Dry eyes | TMH: OD 0.19 mm/ OS 0.15mm, Schirmer’s I test: OD 7mm/5min, OS 4mm/5min, ANA 1:320, SSA/Ro52 (+), hyperimmunoglobulinaemia, thrombocytopenia  LGS (+), SSB (+) |
| LSG: labial gland; FS: focus score; ANA: anti-nuclear antibodies; SSA/Ro52: anti-Ro52/SSA antibodies; SSA/Ro60: anti-Ro60/SSA antibodies; SSB: anti-Ro/SSB antibodies; RF: rheumatoid factor; Tear meniscus height: TMH; Tear film break-up time: BUT | | | | |

| **Supplemental Table S2.** Analysis of factors associated with thrombocytopenia among C-pSS patients. | | | | | | |
| --- | --- | --- | --- | --- | --- | --- |
|  | Univariate analysis | | *P*-Value | Multivariate analysis | | *P*-Value |
|  | OR | 95%CI |  | OR | 95%CI |  |
| ANA |  |  | 0.269 |  |  | 0.482 |
| <1:320 | 1 |  |  | 1 |  |  |
| ≥1:320 | 0.506 | 0.151-1.695 |  | 2.018 | 0.285-14.290 |  |
| SSA/Ro52 |  |  | 0.907 |  |  | 0.405 |
| - | 1 |  |  | 1 |  |  |
| + | 1.101 | 0.219-5.541 |  | 2.766 | 0.252-30.302 |  |
| SSA/Ro60 |  |  | 0.165 |  |  | 0.775 |
| - | 1 |  |  | 1 |  |  |
| + | 0.398 | 0.108-1.464 |  | 1.387 | 0.146-13.142 |  |
| SSB |  |  | 0.004 |  |  | **0.010** |
| - | 1 |  |  | 1 |  |  |
| + | 0.093 | 0.018-0.478 |  | 0.028 | 0.002-0.427 |  |
| RF |  |  | 0.004 |  |  | **0.004** |
| - | 1 |  |  | 1 |  |  |
| + | 0.041 | 0.005-0.353 |  | 0.020 | 0.001-0.291 |  |
| ANA: anti-nuclear antibodies; SSA/Ro52: anti-Ro52/SSA antibodies; SSA/Ro60: anti-Ro60/SSA antibodies; SSB: anti-Ro/SSB antibodies; RF: rheumatoid factor. | | | | | | |

| **Supplemental Table S3.** Analysis of factors associated with parotitis among C-pSS patients. | | | | | | |
| --- | --- | --- | --- | --- | --- | --- |
|  | Univariate analysis | | *P*-Value | Multivariate analysis | | *P*-Value |
|  | OR | 95%CI |  | OR | 95%CI |  |
| ANA |  |  | 0.019 |  |  | 0.318 |
| <1:320 | 1 |  |  | 1 |  |  |
| ≥1:320 | 8.000 | 1.409-45.407 |  | 4.365 | 0.242-78.797 |  |
| SSB |  |  | 0.004 |  |  | **0.012** |
| - | 1 |  |  | 1 |  |  |
| + | 26.714 | 2.877-248.023 |  | 30.518 | 2.102-443.006 |  |
| RF |  |  | 0.004 |  |  | **0.028** |
| - | 1 |  |  | 1 |  |  |
| + | 28.33 | 2.967-270.574 |  | 20.451 | 1.393-300.207 |  |
| ANA: anti-nuclear antibodies; SSA/Ro52: anti-Ro52/SSA antibodies; SSA/Ro60: anti-Ro60/SSA antibodies; SSB: anti-Ro/SSB antibodies; RF: rheumatoid factor. | | | | | | |

| **Supplemental Table S4.** Comparison of immunological markers and organ involvement | | | | | | | | | | | | | | | | | |
| --- | --- | --- | --- | --- | --- | --- | --- | --- | --- | --- | --- | --- | --- | --- | --- | --- | --- |
| Parameters | | Organ involvement (total), % | | Cutaneous involvement, % | | Hematology involvement, % | | Digestive system involvement, % | | Musculoskeletal system involvement, % | | CNS system involvement, % | | Renal involvement, % | | Lung involvement, % | |
| RF + | | 81.3 (13/16) | | 56.3 (9/16) | | 25.0 (4/16) | | 6.3 (1/16) | | 12.5 (2/16) | | 6.3 (1/16) | | 0 (0/16) | | 7.1 (1/14) | |
| RF - | | 86.2 (25/29) | | 48.3 (14/29) | | 75.9 (22/29) | | 10.3 (3/29) | | 0 (0/29) | | 0 (0/29) | | 6.9 (2/29) | | 15.4 (4/26) | |
| *P*-Value | | 0.992 | | 0.608 | | **0.003** | | 1.000 | | 0.121 | | 0.356 | | 0.531 | | 0.802 | |
| ANA <320 | | 75.0 (12/16) | | 50.0 (8/16) | | 75.0 (12/16) | | 6.3 (1/16) | | 0 (0/16) | | 0 (0/16) | | 0 (0/16) | | 6.3 (1/16) | |
| ANA≥320 | | 90.9 (30/33) | | 54.5 (18/33) | | 51.5 (17/33) | | 15.2 (5/33) | | 6.1 (2/33) | | 3.0 (1/33) | | 6.1 (2/33) | | 14.8 (4/27) | |
| *P*-Value | | 0.290 | | 0.765 | | 0.208 | | 0.670 | | 1.000 | | 1.000 | | 1.000 | | 0.723 | |
| SSA/Ro52+ | | 90.5 (38/42) | | 57.1 (24/42) | | 59.5 (25/42) | | 14.3 (6/42) | | 4.8 (2/42) | | 2.4 (1/42) | | 4.8 (2/42) | | 13.5 (5/37) | |
| SSA/Ro52 - | | 57.1 (4/7) | | 28.6 (2/7) | | 57.1 (4/7) | | 0 (0/7) | | 0 (0/7) | | 0 (0/7) | | 0 (0/7) | | 0 (0/6) | |
| P-Value | | 0.080 | | 0.321 | | 1.000 | | 0.574 | | 1.000 | | 1.000 | | 1.000 | | 1.000 | |
| SSA/Ro60+ | | 88.9 (32/36) | | 55.6 (20/36) | | 52.8 (19/36) | | 13.9 (5/36) | | 5.6 (2/36) | | 2.8 (1/36) | | 2.8 (1/36) | | 13.3 (4/30) | |
| SSA/Ro60- | | 76.9 (10/13) | | 46.2 (6/13) | | 76.9 (10/13) | | 7.7 (1/13) | | 0 (0/13) | | 0 (0/13) | | 7.7 (1/13) | | 7.7 (1/13) | |
| *P*-Value | | 0.552 | | 0.560 | | 0.234 | | 0.928 | | 1.000 | | 1.000 | | 1.000 | | 0.990 | |
| SSB + | | 87.5 (14/16) | | 56.3 (9/16) | | 43.8 (7/16) | | 18.8 (3/16) | | 0 (0/16) | | 0 (0/16) | | 6.3 (1/16) | | 14.3 (2/14) | |
| SSB- | | 84.8 (28/33) | | 51.5 (17/33) | | 66.7 (22/33) | | 9.1 (3/33) | | 6.1 (2/33) | | 3.0 (1/33) | | 3.0 (1/33) | | 10.3 (3/29) | |
| *P*-Value | | 1.000 | | 0.755 | | 0.126 | | 0.615 | | 1.000 | | 1.000 | | 1.000 | | 1.000 | |
| LSG: labial gland; FS: focus score; ANA: anti-nuclear antibodies; SSA/Ro52: anti-Ro52/SSA antibodies; SSA/Ro60: anti-Ro60/SSA antibodies; SSB: anti-Ro/SSB antibodies; RF: rheumatoid factor. | | | | | | | | | | | | | | | | | |

| **Supplemental Table S5**. Detailed association of clinical and laboratory features with positive RF and ANA titre≥1:320 | | | | | | |
| --- | --- | --- | --- | --- | --- | --- |
| Parameters | Positive RF | Negative RF | *P*-Value | ANA titre <1:320 | ANA titre ≥1:320 | *P*-Value |
| Age (years), mean ± SD | 10.08 ±3.23 | 10.71 ±3.42 | 0.552 | 10.55±3.45 | 10.23±3.50 | 0.763 |
| Dry eyes, % | 25.0 (4/16) | 17.2 (5/29) | 0.815 | 18.8 (3/16) | 18.2 (6/33) | 1.000 |
| Dry mouth, % | 18.8 (3/16) | 20.7 (6/29) | 1.000 | 18.8 (3/16) | 18.2 (6/33) | 1.000 |
| Dental caries, % | 25.0 (4/16) | 27.6 (8/29) | 1.000 | 25.0 (4/16) | 30.3 (10/33) | 0.962 |
| Parotitis, % | 90.9 (10/11) | 26.1 (6/23) | **0.001** | 18.2 (2/11) | 64.0 (16/25) | **0.027** |
| Raynaud’s phenomenon, % | 12.5 (2/16) | 3.4 (1/29) | 0.589 | 0 (0/16) | 9.1 (3/33) | 0.541 |
| Arthritis or arthralgia, % | 25.0 (4/16) | 27.6 (8/29) | 1.000 | 25.0 (4/16) | 27.3 (9/33) | 1.000 |
| Fever, % | 37.5 (6/16) | 24.1 (7/29) | 0.344 | 31.3 (5/16) | 27.3 (9/33) | 0.773 |
| Schirmer’s I test positive, % | 28.6 (4/14) | 55.6 (15/27) | 0.189 | 53.8 (7/13) | 43.3 (13/30) | 0.526 |
| LSG biopsy performed |  |  | 0.195 |  |  | 0.433 |
| LSG biopsy FS 1-4, % | 55.6 (5/9) | 81.0 (17/21) |  | 83.3 (10/12) | 68.4 (13/19) |  |
| LSG biopsy ≥5, % | 44.4 (4/9) | 19.0 (4/21) |  | 16.7 (2/12) | 31.6 (6/19) |  |
| IgG (g/L) | 17.52±5.81 | 15.36±6.31 | 0.181 | 17.27±7.03 | 16.56±7.31 | 0.758 |
| IgA (g/L) | 2.18±0.95 | 1.78±0.72 | 0.130 | 1.89±0.85 | 1.90±0.79 | 0.961 |
| IgM (g/L) | 1.46±0.68 | 1.09±0.39 | **0.044** | 1.26±0.59 | 1.14±0.37 | 0.819 |
| C3 (g/L) | 1.15±0.34 | 1.20±0.21 | 0.141 | 1.18±0.26 | 1.16±0.28 | 0.689 |
| C4 (g/L) | 0.24±0.10 | 0.19±0.07 | 0.195 | 0.18±0.09 | 0.21±0.08 | 0.125 |
| CH50 (U/mL) | 37.50±14.30 | 39.74±12.99 | 0.443 | 39.33±16.27 | 38.31±11.32 | 0.522 |
| ALT (U/L) | 26.06±17.37 | 33.83±25.34 | 0.434 | 30.38±19.58 | 33.67±28.80 | 0.974 |
| AST (U/L) | 31.13±15.33 | 31.93±14.64 | 0.812 | 29.69±11.51 | 33.45±17.45 | 0.536 |
| Globulin (g/L) | 34.78±5.27 | 33.10±6.37 | 0.231 | 33.64±4.09 | 34.57±7.30 | 0.915 |
| WBC (×10^9^/L) | 6.06±1.95 | 7.65±3.15 | 0.075 | 6.86±2.67 | 6.98±3.00 | 0.895 |
| Hb (g/L) | 121.75±14.66 | 123.48±21.95 | 0.400 | 122.69±20.55 | 122.52±18.09 | 0.814 |
| PLT(×10^9^/L) | 277.44±81.09 | 170.17±181.07 | **0.030** | 143.06±157.10 | 226.36±156.04 | 0.087 |
| Cr (μmol/L) | 40.25±7.25 | 42.62±10.60 | 0.399 | 43.31±12.03 | 40.27±8.63 | 0.468 |
| BUN (mmol/L) | 4.18±0.81 | 4.65±0.92 | 0.085 | 4.79±1.01 | 4.41±0.84 | 0.186 |
| ESR (mm/h) | 27.67±31.89 | 19.93±13.67 | 0.120 | 16.33±11.22 | 21.35±25.52 | 0.963 |
| Hyperglobulin, % | 81.3 (13/16) | 42.9 (12/28) | **0.031** | 68.8 (11/16) | 57.6 (19/33) | 0.452 |
| Hyperimmunoglobulinaemia, % | 40 (6/15) | 50.0 (14/28) | 0.531 | 40.0 (6/15) | 46.9 (15/32) | 0.899 |
| LSG: labial gland; FS: focus score; ANA: anti-nuclear antibodies; SSA/Ro52: anti-Ro52/SSA antibodies; SSA/Ro60: anti-Ro60/SSA antibodies; SSB: anti-Ro/SSB antibodies; RF: rheumatoid factor; C3: complement 3; C4: complement 4; CH50: complement total activity; IgG: immunoglobulin G; IgA: immunoglobulin A; IgM: immunoglobulin M; ESR: erythrocyte sedimentation rate; WBC: white blood cells; Hb: hemoglobin; PLT: platelets; BUN: urea nitrogen; ALT: alanine aminotransferase; AST: aspartate aminotransferase; Cr: creatinine; ESR: erythrocyte sedimentation rate. | | | | | | |

| **Supplemental Table S6**. Detailed association of clinical and laboratory features with positive SSA/Ro52, positive SSA/Ro60, and positive SSB | | | | | | | | | | |
| --- | --- | --- | --- | --- | --- | --- | --- | --- | --- | --- |
| Parameters | SSA/Ro52+ | SSA/Ro52- | | *P*-Value | SSA/Ro60+ | SSA/60- | *P*-Value | SSB+ | SSB- | *P*-Value |
| Age (years), mean ± SD | 10.14±3.42 | 11.55±3.71 | | 0.323 | 10.41±3.33 | 10.15±3.92 | 0.823 | 10.47±2.83 | 10.28±3.76 | 0.858 |
| Dry eyes, % | 11.9 (5/42) | | 57.1 (4/7) | **0.020** | 11.1 (4/36) | 38.5 (5/13) | 0.078 | 31.3 (5/16) | 12.1 (4/33) | 0.219 |
| Dry mouth, % | 19.0 (8/42) | 14.3 (1/7) | | 1.000 | 19.4 (7/36) | 15.4 (2/13) | 1.000 | 25.0 (4/16) | 15.2 (5/33) | 0.659 |
| Dental caries, % | 26.2 (11/42) | 42.9 (3/7) | | 0.651 | 27.8 (10/36) | 30.8 (4/13) | 1.000 | 18.8 (3/16) | 33.3 (11/33) | 0.470 |
| Parotitis, % | 56.3 (18/32) | 0 (0/4) | | 0.104 | 64.3 (18/28) | 0 (0/8) | **0.003** | 91.7 (11/12) | 29.2 (7/24) | **0.001** |
| Raynaud’s phenomenon, % | 7.1 (3/42) | 0 (0/7) | | 1.000 | 5.6 (2/36) | 7.7 (1/13) | 1.000 | 12.5 (2/16) | 3.0 (1/33) | 0.508 |
| Arthritis or arthralgia, % | 28.6 (12/42) | 14.3 (1/7) | | 0.741 | 27.8 (10/36) | 23.1 (3/13) | 1.000 | 12.5 (2/16) | 33.3 (11/33) | 0.229 |
| Fever, % | 28.6 (12/42) | 28.6 (2/7) | | 1.000 | 30.6 (11/36) | 23.1 (3/13) | 0.878 | 25.0 (4/16) | 30.3 (10/33) | 0.962 |
| Schirmer’s I test positive, % | 45.9 (17/37) | 50.0 (3/6) | | 1.000 | 43.8 (14/32) | 54.5 (6/11) | 0.536 | 57.1 (8/14) | 55.2 (16/29) | 1.000 |
| LSG biopsy performed |  |  | | 0.291 |  |  | 0.076 |  |  | 0.154 |
| LSG biopsy FS 1-4, % | 69.2 (18/26) | 100.0 (5/5) | |  | 65.2 (15/23) | 100 (8/8) |  | 50.0 (4/8) | 82.6 (19/23) |  |
| LSG biopsy ≥5, % | 30.8 (8/26) | 0 (0/5) | |  | 34.8 (8/23) | 0 (0/8) |  | 50.0 (4/8) | 17.4 (4/23) |  |
| IgG (g/L) | 17.80±7.14 | 11.88±3.62 | | **0.048** | 18.62±7.32 | 12.43±3.37 | **0.007** | 19.23±6.84 | 16.01±7.02 | 0.091 |
| IgA (g/L) | 1.89±0.84 | 1.87±0.74 | | 0.943 | 2.00±0.82 | 1.58±0.79 | 0.135 | 2.12±0.71 | 1.79±0.86 | 0.203 |
| IgM (g/L) | 1.25±0.55 | 1.01±0.33 | | 0.503 | 1.27±0.54 | 1.07±0.47 | 0.283 | 1.35±0.63 | 1.16±0.47 | 0.273 |
| C3 (g/L) | 1.16±0.28 | 1.18±0.14 | | 0.491 | 1.14±0.27 | 1.22±0.27 | 0.360 | 1.20±0.35 | 1.15±0.23 | 0.802 |
| C4 (g/L) | 0.21±0.09 | 0.16±0.03 | | 0.232 | 0.19±0.09 | 0.21±0.09 | 0.467 | 0.25±0.10 | 0.18±0.07 | **0.008** |
| CH50 (U/mL) | 37.08±13.25 | 47.57±7.32 | | **0.006** | 37.47±13.82 | 41.69±10.64 | 0.146 | 39.20±15.11 | 38.38±12.21 | 0.819 |
| ALT (U/L) | 32.79±26.87 | 31.43±21.63 | | 0.791 | 32.85±23.21 | 32.85±23.21 | 0.955 | 37.19±36.28 | 30.36±19.51 | 0.839 |
| AST (U/L) | 32.69±16.43 | 29.43±11.15 | | 0.748 | 32.00±16.67 | 32.85±13.38 | 0.870 | 36.44±20.91 | 30.18±12.37 | 0.405 |
| Globulin (g/L) | 34.64±6.37 | 32.00±6.58 | | 0.332 | 35.28±6.60 | 31.45±4.99 | 0.056 | 36.78±7.58 | 33.05±5.46 | 0.107 |
| WBC (×10^9^/L) | 6.76±2.85 | 8.03±2.93 | | 0.283 | 6.96±2.92 | 6.87±2.83 | 0.926 | 6.16±1.95 | 7.31±3.18 | 0.190 |
| Hb (g/L) | 119.66±18.52 | 136.57±12.27 | | **0.022** | 119.44±18.88 | 131.23±15.81 | **0.029** | 117.63±22.65 | 124.97±16.32 | 0.387 |
| PLT(×10^9^/L) | 202.50±166.02 | 179.14±122.98 | | 0.724 | 205.64±157.81 | 181.23±169.99 | 0.642 | 287.94±146.41 | 156.12±149.51 | **0.005** |
| Cr ((μmol/L) | 40.12±8.53 | 48.14±14.60 | | **0.045** | 40.86±8.39 | 42.38±13.43 | 0.637 | 39.75±7.91 | 42.00±10.69 | 0.459 |
| BUN (mmol/L) | 4.57±0.90 | 4.30±0.94 | | 0.467 | 4.56±0.92 | 4.45±0.90 | 0.716 | 4.22±0.76 | 4.68±0.94 | 0.092 |
| ESR (mm/h) | 21.79±23.13 | 8.14±4.45 | | 0.071 | 23.18±24.69 | 10.92±7.34 | 0.110 | 28.87±32.17 | 15.29±13.16 | 0.119 |
| Hyperglobulin, % | 61.9 (26/42) | 57.1 (4/7) | | 1.000 | 69.4 (25/36) | 38.5 (5/13) | **0.049** | 75.0 (12/16) | 54.5 (18/33) | 0.287 |
| Hyperimmunoglobulinaemia, % | 48.8 (20/41) | 16.7 (1/6) | | 0.299 | 48.6 (17/35) | 33.3 (4/12) | 0.562 | 60.0 (9/15) | 37.5 (12/32) | 0.148 |
| LSG: labial gland; FS: focus score; ANA: anti-nuclear antibodies; SSA/Ro52: anti-Ro52/SSA antibodies; SSA/Ro60: anti-Ro60/SSA antibodies; SSB: anti-Ro/SSB antibodies; RF: rheumatoid factor; C3: complement 3; C4: complement 4; CH50: complement total activity; IgG: immunoglobulin G; IgA: immunoglobulin A; IgM: immunoglobulin M; ESR: erythrocyte sedimentation rate; WBC: white blood cells; Hb: hemoglobin; PLT: platelets; BUN: urea nitrogen; ALT: alanine aminotransferase; AST: aspartate aminotransferase; Cr: creatinine; ESR: erythrocyte sedimentation rate. | | | | | | | | | | |
